# Patients’ experiences treated with open-label placebo versus double-blind placebo: a mixed methods qualitative study

**DOI:** 10.1186/s40359-022-00731-w

**Published:** 2022-02-04

**Authors:** Julia W. Haas, Giulio Ongaro, Eric Jacobson, Lisa A. Conboy, Judy Nee, Johanna Iturrino, Vikram Rangan, Anthony Lembo, Ted J. Kaptchuk, Sarah Ballou

**Affiliations:** 1 Division of Gastroenterology, Beth Israel Deaconess Medical Center/Harvard Medical School, 330 Brookline Ave, Boston, MA 02215 USA; 2 Program in Placebo Studies, Beth Israel Deaconess Medical Center/Harvard Medical School, Boston, MA USA; 3 Department of Anthropology, The London School of Economics and Political Science, London, UK; 4 Department of Global Health and Social Medicine, Harvard Medical School, Boston, MA USA

**Keywords:** Placebo effect, Open-label placebo, Irritable bowel syndrome, Qualitative research

## Abstract

**Background:**

There is increasing evidence suggesting that open-label placebo (OLP) is an effective treatment for several medical conditions defined by self-report. However, little is known about patients’ experiences with OLP, and no studies have directly compared patients’ experiences in double-blind placebo (DBP) conditions.

**Methods:**

This study was nested in a large randomized-controlled trial comparing the effects of OLP and DBP treatments in individuals with irritable bowel syndrome (IBS). We randomly selected 33 participants for interviews concerning their experiences in the parent trial. The data were qualitatively analyzed using an iterative immersion/crystallization approach. We then compared the qualitative interview data to the quantitative IBS severity data assessed during the parent trial, using a mixed methods approach.

**Results:**

Two prominent interview themes were identified: (1) the participants’ feelings about their treatment allocation and (2) their reflections about the treatment. Both OLP and DBP participants mentioned hope and curiosity as major feelings driving them to engage with their treatment. However, while DBP participants tended to be more enthusiastic about their allocation, OLP participants were more ambivalent. Furthermore, OLP participants reflected more on their treatment, often involving noticeable cognitive and emotional processes of self-reflection. They offered a variety of explanations for their symptom improvement and were significantly less likely to attribute it to the treatment itself than DBP participants (*Χ*^2^ [3] = 8.28; *p* = .041). Similarly, the participants’ retrospective narratives of symptom improvement were significantly correlated with their corresponding quantitative IBS severity scores only in DBP (*p*’s ≤ .006) but not in OLP (*p*’s ≥ .637).

**Conclusion:**

OLP and DBP participants share feelings of hope, uncertainty and curiosity but differ in the extent of conscious reflection. The counter-intuitive OLP prompts more self-examination, ambivalent feelings and active engagement compared to DBP. At the same time, OLP participants are more reluctant to attribute symptom improvement to their treatment. Our findings substantially add to the emerging picture of factors that distinguish OLP and DBP and their potential mechanisms.

## Introduction

Placebo *effects* are the salubrious clinical outcomes patients receive from immersion in the rituals, symbols, and behaviors of medical treatment. Placebo *responses* are clinical improvements due to placebo effects plus other non-specific factors such as spontaneous improvement, natural fluctuations and regression to the mean [1, 2]. In recent years, there has been a burst of quantitative clinical and basic science research investigating placebo effects [3–7]. The use of qualitative methods to assess patients’ experience with placebo treatment has been scarce and confined to double-blind placebo (DBP) administration [8]. To our knowledge, there are no published qualitative studies of patients’ experience in open-label placebo (OLP), nor any studies that compare the experience of OLP to DBP. This study seeks to address this lacuna and offer an account of the differences in how patients experience these two treatments.

Until recently, conventional medical thinking assumed that placebo treatments only elicit placebo effects when administered deceptively or as part of double-blind randomized controlled trials (RCTs). Reflecting conventional wisdom, Henry Beecher, the famous Harvard anaesthesiologist who helped to pioneer placebo studies, maintained that a placebo pill only works “as long as it is not detected as a placebo by the subject or the observer” so that the patient “believes it [is a drug] and consequently the expected results occur” [9]. In 2010, our team performed the first RCT to challenge this widespread belief. We found that when participants were given “open-label”, i.e., honestly prescribed placebos (OLP) for irritable bowel syndrome (IBS) on top of their treatment as usual (TAU) they had significant and clinical meaningful improvement compared to the control group of TAU alone [10]. The TAU group controlled for spontaneous improvement, the patient–physician relationship, and regression to the mean which allowed the inference that the improvement were actual placebo effects and not due to other non-specific factors. Our original RCT was followed by a flurry of OLP RCTs. Recently, a meta-analysis of thirteen OLP RCTs—including conditions such as episodic migraine attacks, chronic low back pain, cancer-related fatigue and menopausal hot flashes—demonstrated that OLP can produce significant effects (standardized mean difference = 0.72; 95% CI 0.39–1.08; *p* < 0.0001) [11]. OLP has now become a subject of inquiry as much as double-blind placebo (DBP). To date, however, no study has investigated the qualitative experiences of patients on OLP or compared them to those of DBP patients.

Recently, our team completed a large RCT (N = 308) that sought to replicate our earlier finding of OLP + TAU versus TAU alone in irritable bowel syndrome (IBS) [12]. IBS is a common functional gastrointestinal disorder characterized by abdominal pain accompanied by diarrhea or constipation. It is a very common condition affecting 4.1% of the worldwide population [13]. Like many other functional disorders, IBS is highly susceptible to psychosocial factors and shows high placebo responses in clinical trials. Our parent RCT replicated the 2010 findings and confirmed that OLP was superior to TAU (*p* = 0.031), evoking clinical meaningful benefits in IBS. An additional aim of the parent trial was to determine whether the magnitude of OLP and DBP were similar or different. To that end, our parent RCT nested a double-blind RCT comparison of DBP and double-blind peppermint oil into the original design of OLP versus TAU. Participants were told that they could be randomized to OLP, a double-blind RCT where the pill could be either placebo or peppermint oil, or TAU. The nested comparison of OLP versus DBP demonstrated, to our knowledge, for the first time in history, that symptom improvement, at least in IBS, was not significantly different in OLP and DBP (*p* = 0.485) [12].

We nested our qualitative study in this parent RCT described above. Nesting our interviews in the large parent study not only allowed us to directly compare the experiences of participants randomized to OLP to the experiences of participants allocated to DBP, but also to compare both of these to the few existing qualitative studies of patients on blinded placebo [8, 14]. Furthermore, we were able to include quantitative data of the parent trial into our qualitative analyses to present mixed methods findings of participants’ experiences on OLP and DBP.

## Methods

This study was embedded in a large, six-week RCT which investigated the effects of OLP and DBP on symptom severity in individuals with IBS. The full methodology of the parent study is reported in detail in the previously published study protocol [15] as well as the publication of the major findings [12]. Ethical and regulatory approvals were obtained from the Institutional Review Board for the Protection of Human Subjects at Beth Israel Deaconess Medical Center. Our study was performed and reported in accordance with the relevant guidelines and regulations, informed by COREQ [16].

### Interviews

At the beginning of the parent trial, 33 participants were randomly assigned to be individually interviewed after completion of the quantitative study. Interviewed participants were either from the OLP group (n = 11) or from the double-blind condition (n = 17), which included both double-blind placebo and double-blind peppermint participants. However, the double-blind participants were unaware of their group assignment, and symptom improvement did not differ between placebo and peppermint participants [17]. Therefore, we do not distinguish between the two double-blind groups in this paper and we classify them both as DBP participants. Experimenters in the DBP group were also blinded to treatment assignment. In all groups, outcome assessments were performed by blinded research assistants. Five TAU participants were interviewed as well but are not included in these analyses as we were only interested in patients’ experiences with placebo treatments. Due to equipment malfunction, the interviews of three participants of the double-blind condition were not audio taped, resulting in a final sample of N = 25.

The interviewers were experienced qualitative researchers of our team (EJ, LC, and SB) who were not otherwise involved in study procedures. Each interview took approximately 30 to 45 min and included semi-structured questions about the participants’ feelings, thoughts and behaviors during the course of the six-week trial, any changes they experienced, as well as their overall impression of the study. A selected sample of the most important questions is provided in Table 1. All interviewed participants had given written consent to be interviewed and audio recorded. The recordings were anonymous, i.e. no personal information was included in the later transcribed audio files.

**Table 1:** Selected sample of most relevant interview questions

How did you think about your IBS when you first joined the study? How did you think about placebos? How did you think about peppermint oil?
Please tell me the story of how the study has gone for you What study experiences stand out the most in your memory?
How did your first meeting with the doctor go? • What do you remember? • What group were you assigned to? • How did the doctor explain it? • How did you feel about your assignment? • Did you prefer a different group?
Did you notice any effects from taking the pills? If yes: • When did you notice those effects? • How did the effects go from there? • How do you think taking the pill caused those effects? If no: • Why do you think there weren’t any effects?
What changed during the time you were in the study? • When did you notice those changes? • How did the changes go over time? • What do you think caused the changes?
Has being in the study changed how you think about placebos? Has being in the study changed how you think about peppermint oil? Has being in the study changed how you think about your IBS?
How do you think your IBS will go in the future?

Bullet point questions are prompts that were only asked if needed

IBS, irritable bowel syndrome

### Placebos and other treatments

The placebo pills consisted of 0.2 ml enteric coated soybean oil softgels. The peppermint oil was also dispensed in enteric coated pills, which for all practical purposes eliminated smell and taste as to prevent patients to identify their assignment. All patients were allowed to continue with their treatment as usual and agreed not to change IBS medications or doses during the 6 weeks of the trial.

### Qualitative analysis

We employed an iterative immersion/crystallization approach [18] to qualitatively analyze the interview transcripts. Two authors (JH and GO) read the interviews independently and identified the major themes, which were then refined through several group discussions by the entire team. This paper focuses on the two most prominent themes that emerged, and we have organized the qualitative results according to these two themes: (1) feelings about treatment allocation, and (2) reflections about the treatment. In each section, we first present commonalities between the two groups and then identify the differences.

### Relation of qualitative and quantitative data

To compare our qualitative data to the quantitative results of the parent trial, we also adopted a mixed methods approach. In order to make our report more readable, we will present the methodology of our mixed methods section after the qualitative results and right before the mixed methods results.

## Results

### Feelings about treatment allocation

#### Similarities

##### **Preference**

When the participants were asked how they felt about their group assignment and which treatment group they had preferred, the overall reactions were similar in DBP and OLP participants. The majority were pleased with their assignment and felt lucky that they had not been randomized to the no treatment control group.

*250 (OLP):* *I was actually happy about that, because I know that one of the groups was going to be taking, and doing, nothing. And I didn’t wanna be in that group, [...] because I felt like that was just gonna do nothing for me (laughs).*
*309 (DBP):* *I was glad that I was assigned to take a pill instead of just being the control and not doing anything.*

##### **Hope**

In both treatment groups, hope seemed to play an important role. Without being prompted, many participants said they were hopeful about their treatment.

*184 (OLP):* *I was hoping that it would help.*
*187 (DBP):* *I was hopeful. I was really hopeful.*
*143 (DBP):* *I think I was hoping to, I was hoping to get something that would make me feel better.*
*138 (OLP):* *I was excited and, again, hopeful that it would work for me.*

##### **Curiosity**

Besides hope, curiosity was another unprompted feeling mentioned by several participants of both groups:

*149 (OLP):* *I was just curious, honestly.*
*143 (DBP) :* *I was just curious to see how it would, how it would unroll.*
*185 (OLP):* *I had an open mind about it. I didn’t think that it definitely would work or that it definitely wouldn’t. Um, but, yeah, I was just curious, um, and, yeah, I guess hopeful that there would be a positive effect.*

#### Differences

##### **Expression of feelings**

Despite the overall homogeneity in the participants’ attitudes about their treatment, there were prominent group differences in the way they described their feelings about their group assignment. It seemed that many participants preferred the clarity of DBP to the paradoxical idea of taking an “inert” substance.

##### **Enthusiasm in DBP**

In the DBP group, participants appeared to express more positive feelings. The majority used words like “happy”, “thankful”, “thrilled”, “excited”, or “intrigued”, and they often emphasized these feelings with “very” or “really”. Most of them stated clearly that they wanted to be in the DBP group and that they were very glad about actually having been assigned to it.

*134 (DBP):* *Um, it made me feel really good that I was gonna be able to try something that may help me. […] I was very happy with it. […] I’m glad I was in the group I was in. You know, after the first week, I was thrilled.*
*139 (DBP):* *They told me I’d be getting the pill that may or may not be a placebo. […] Which I was thankful for. […] I really wanted to get in the one that would be either-or.*
*187 (DBP):* *I was grateful for being in the double-blind study. […] I was intrigued to find out what would happen. […] I was thrilled.*
*230 (DBP):* *I was hoping I would be randomized to the arm that would have the peppermint oil. […] This is the arm I wanted (laughing). So, it worked out well.*
*311 (DBP):* *And I think the double-blind almost makes it like more, more fun.*

##### **Ambivalence in OLP**

In the OLP group, participants often expressed ambivalent feelings or a neutral response. Higher uncertainty about the treatment was evident in the frequent use of wordings such as “kind of”, “maybe”, “I guess”, or “a little,” and a hesitancy to make judgements was noticeable. Several OLP participants conceded they had initially preferred the double-blind group but emphasized their open mind toward OLP. Others said that they were indifferent about their randomization.

*174 (OLP):* *Um, and I remember really having a very neutral response […] and not being disappointed or, you know, upset or any way about it.*
*184 (OLP):* *I was hoping to get the peppermint oil. […] I was a little skeptical about the placebo, but I do believe there’s a mind–body connection […] So I was open to the placebo. […] I was open to it.*
*091 (OLP):* *Um, I had no […] no attitude.*
*138 (OLP):* *No, I didn’t have a preference.*
*149 (OLP):* *I kind of [had a preference], especially after he had talked about the effects of peppermint oil […] And it was sort of like it just clicked. I was like, of course, that would be a good alternative, because I drink peppermint tea. So, I kind of wanted to be on that one.*
*271 (OLP):* *I don’t know if I did [have a preference]. Eh, I’m not sure. […] I think I probably would have preferred the blinded one.*

### Reflections about the treatment

#### Similarities

##### **Potential power of the placebo effect**

Most participants in both groups had heard or were aware of the “potential power of the placebo effect,” and they accepted that it could have played a role for their personal symptom improvement during the study. There were both DBP and OLP participants who considered placebo effects to be the most likely explanation for their symptom improvement.

*217 (DBP):* *I think it depends a lot on whether it’s a placebo or not. Or maybe it doesn’t—like maybe the placebo effect is strong and so the peppermint oil is actually doing nothing, and it’s the placebo effect anyway.*
*184 (OLP):* *Taking what I thought was a medication—you know, it would, it was helping me, yeah.*
*187 (DBP):* *Well, if it’s the peppermint oil then I don’t know much how it works, except that it calms the gut. Um, but I purposely did not go online and read about it. So, and if it’s the, um, the placebo then again, that’s the brain–gut connection, and I’m happy with that.*

For a few participants, the conversation about placebo effects naturally developed into a discussion of “mind–body connection” or “mindfulness.”

*311 (DBP)*: *It’s interesting to me that you know we’re getting to this point in medicine where maybe we’re realizing that we need to like back off of certain medications and instead talk about mindfulness and um good habits that sort of set our bodies up to perform better, I guess.*
*112 (OLP)*: *And I do believe in my heart anyway, I’m a yoga person and I do believe there is a giant mind-body connection, so, um, I have no problem taking something, again, that’s not a chemical that will help me. […] [The idea of taking placebo] actually almost reinforced to me that there’s definitely a mind-body connection and possibly that I could also heal a little bit like almost like self-healing.*

#### Differences

##### **Causal attribution**

Despite the broad acknowledgment of the power of placebo effects across groups, the attribution of cause to personal improvement (or lack thereof) varied between OLP and DBP participants. We found that both narratives (with the exception of participants who did not feel any improvement) were replete with feelings of uncertainty about the outcome and nature of treatment, but that this uncertainty was expressed differently depending on the group. Participant 112 had a hypothesis similar to our findings:

*112 (OLP)*: *[If I would have been in the DBP group,] I probably would've been questioning more in my mind, [why] am I feeling symptoms now […]? I think there would’ve been more thinking involved on my part [and] almost anxiety of which one is it and how do my symptoms fit in with that actual placebo or non-placebo […] If I hadn’t known I would just probably attribute some of my symptoms differently.*

##### **Attribution in DBP—passive engagement with treatment**

One of the main aspects that distinguishes the experience of DBP participants from that of the OLP participants is the guesswork that the former often engaged in while taking part in the trial (and which is of course missing for OLP participants). This consisted in trying to find cues that would indicate whether they had been assigned to the placebo or peppermint arm. Beyond this type of guesswork, however, we found that DBP patients were less actively engaged with the treatment than OLP patients, who, as we show below, consciously tried to come to terms with the novelty and counter-intuitive, paradoxical nature of OLP. In DBP, if a participant felt better, they endorsed the proposition that they had received peppermint oil. If they felt no difference, they gave more thought to the possibility of having received placebo.

*214 (DBP):* *I, I think it worked for me, and I, I found that I have a distinct feeling I took the peppermint oil. […] And it worked.*
*309 (DBP):* *I’m assuming that I, having the peppermint, um it just it makes sense that, that would’ve calmed things down.*
*230 (DBP):* *I would say after the first visit or the second visit, which was at the halfway mark, I was kind of on the fence, I could have gone either way if I had the placebo or the, um, peppermint oil. But then it wasn’t until this last week that I really felt like […] you know, like I do think it could be the peppermint oil.*
*281 (DBP):* *So, I’m like, “I wonder which it is. Is it the placebo effect? Is it my mind over my body? I wonder.”*

##### **Attribution in OLP—active engagement with placebo**

OLP participants were aware from the beginning that they were taking an inert substance. Participants who showed improvement of symptoms after taking OLP offered a variety of explanations. With the exception of two participants who said, ‘I don’t know’, participants advanced cognitive and emotional explanations that ranged from the experience of meeting with the doctors (itself thought to be beneficial),

*184 (OLP):* *I think it was coming here and meeting with the doctors.*

To the ‘power of the mind’ and the existence of a brain-gut connection,

*138 (OLP):* *It could be, like I said, the brain-gut relationship and, and I had, you know, very much bought into that.*
*174 (OLP):* *Sometimes, I, I remember thinking, “Okay, here you are. You’re taking another pill. This is something else that’s sending, you know, your neurons ... You know, firing and this...” You know, so I kind of, like, embraced the idea of, like, I know that this is not medication, but this is something that is maybe gonna send chemicals to my brain and hopefully work a little bit. […] I guess I just saw it as, “If this is something that is doing something to support your, your brain in order to better connect to your gut, then, um, why not try to embrace it?”*

To the idea that the exercise of taking a pill increased their awareness or “mindfulness” of their body,

*256 (OLP):* *Um, you know, it was a mindfulness experience. But it, you know like it really, it sort of made me think. I mean the whole process of having to remember that, “Oh yeah, I have to take the pill because I’m doing this thing,” just made me more aware of how I was feeling.*
*184 (OLP):* *It works because it’s like mind over matter.*

And, finally, to the suggestion that simply the active attempt to change the situation could have an impact.

*174 (OLP):* *I remember saying, “Even if psychologically, I’m doing something to make myself feel better or I’m being proactive, that, that could have an impact”.*

Explanations about the efficacy of OLP that resort to the ‘power of the mind’ were often accompanied by laughter, skepticism or incredulity, as this conversation illustrates:

*250 (OLP):* *At first I was a little skeptical, ‘cause I’m thinking, “Well it’s not really anything that I’m taking.” I know it’s a placebo pill, so it’s not really like... a real drug. So, I was like, „Oh, I wonder if this is really gonna work.” But maybe, just the thought of it, wanting it to work? Maybe that made it work. […] [Now,] I think that [placebos] probably do work. I think that maybe if you put your mind to making it work, it, I mean I know that sounds weird, but, something is going on. […] I don’t know why it works and has helped. I don’t know if it’s just, you know, all in the head, ‘cause you want it to. […]*
*Interviewer:* *You’re attributing your improvement to the placebo pills?*
*250 (OLP):* *Definitely. Yes.*
*Interviewer:* *What makes you think it was the placebo?*
*250 (OLP):* *Uh … I don’t know, I think it’s just in my head that it’s that (laughs).*
*Interviewer:* *Any thoughts about how or why it has helped?*
*250 (OLP):* *Maybe ‘cause I want it to help? (laughs) […] I want it to be it, that’s what is helping me.*

At the same time, some OLP participants thought that placebo effects would not work on themselves but only on other persons, even if their questionnaire scores demonstrated that they had benefitted from the OLP treatment. A reluctance to admit that they could be a placebo responder, with its implication that IBS might be “all in their head” was evident.

*149 (OLP):* *I wouldn’t say I came with a bad attitude, but just a, like, I did not think that the placebo effect would work on me. […] Even though I know it really does work on a lot of people. […] I don’t think I totally fell into that category. […] But just, like I said, really did not expect me to be someone who—or the placebo effect would work […] on me.*

[Note: After the entire study was over, we examined this participant’s individual IBS severity score, and found a dramatic improvement of symptoms.]

While OLP participants who felt their symptoms had improved offered a variety of possible explanations, the minority of OLP participants who did not notice any symptom improvement were quick to attribute failure to the fact that they received something inert.

*138 (OLP):* *Um, because (laughs) there’s not enough of anything in there.*
*149 (OLP):* *I think, just knowing that it was a placebo, and that, typically a placebo shouldn’t do anything. […] I maybe would have been a bit more dedicated, in taking [the pills], if I really thought it was […] like a drug, or something that was like really going to be helping.*
*307 (OLP)*: *Probably because I knew it was a placebo.*

### Relation of qualitative and quantitative data

#### Statistical approach

To compare our qualitative data to the quantitative results of the parent trial, a mixed methods approach was used. By extracting quantitative data on what participants reported about *symptom improvement* and *attribution* from our qualitative data, we created three variables. First, the dichotomous variable *Improvement Yes/No* was created to rate whether participants said their symptoms had improved during the trial (rated as 1) or had not improved (rated as 0). Second, the variable *Degree of Improvement* was created to further specify this subjective improvement into the experience of high (3), medium (2), mild (1) or no (0) improvement. Finally, the variable *Attribution of Improvement* was created to categorize whether participants thought their improvement was certainly due to the treatment (4), rather due to the treatment (3), rather due to other reasons (2) or certainly due to other reasons (1). Participants who said that their symptoms had not improved during the trial were not included in the analysis of this third variable because they could obviously not attribute their improvement to any reasons if they did not realize improvement.

Both in the open-label and in the double-blind condition, the two variables *Improvement Yes/No* and *Degree of Improvement* were correlated using bivariate correlations (Spearman’s rho [*r*_s_]) with corresponding quantitative variables, i.e. the participants’ symptom improvement on the IBS Severity Scoring System (IBS-SSS). The IBS-SSS is a well-established measure of IBS symptoms [19] and served as the primary outcome in the parent trial. A decrease on the IBS-SSS of at least 50 points is considered a clinically meaningful improvement, a decrease of at least 100 points is considered a medium, and a decrease of at least 150 points a high improvement of IBS symptoms. Thus, a pair of quantitative variables (*IBS-SSS Improvement Yes/No* and *IBS-SSS Degree of Improvement*) was created correspondingly to the quantitized interview variables, adopting the same coding schemes to enable direct comparison. To determine quantitatively whether the participants’ *Attribution of Improvement*, as derived from the interviews, was dependent on participants’ treatment condition, a Chi-Square test was calculated.

#### Findings: symptom improvement

We found that qualitative and quantitative data on symptom improvement correlated strongly in DBP participants but not in OLP participants. Tables 2 and 3 present the bivariate Spearman correlations of the corresponding interview and questionnaire variables within both treatment conditions. In the double-blind condition (Table 2), both the two yes/no variables on improvement and the two variables on the degree of improvement correlate strongly (both *r*_s_ ≥ 0.69; both *p*’s ≤ 0.006). This indicates that in this group, the participants’ narrative description of symptom improvement and their more objective IBS-SSS scores are pretty much in line with each other. Interestingly, the corresponding correlations in the open-label condition (Table 3) are only very weak (both *r*_s_ ≤ 0.16; both *p*’s ≥ 0.637), indicating that the verbal self-report of OLP participants differed from their measurements taken from baseline to endpoint with a validated questionnaire.

**Table 2:** Bivariate correlations of symptom improvement variables in the **double-blind** condition

Symptom improvement			IBS-SSS		Interview	
			Yes/No^a^	Degree^b^	Yes/No^c^	Degree^d^
IBS-SSS	Yes/No^a^	Spearman’s rho	1	.658	.782	.580
		*p* value		.011	.001	.030
	Degree^b^	Spearman’s rho	.658	1	.772	.695
		*p* value	.011		.001	.006
Interview	Yes/No^c^	Spearman’s rho	.782	.772	1	742
		*p* value	.001	.001		.002
	Degree^d^	Spearman’s rho	.580	.695	742	1
		*p* value	.030	.006	.002	

Sample size n = 14

IBS-SSS = irritable bowel severity scoring system

^a^“Yes” is specified as at least 50 points of improvement on the IBS-SSS

^b^Degree is specified as no (< 50 points improvement on the IBS-SSS), mild (≥ 50 points improvement), moderate (≥ 100 points improvement) or high (≥ 150 points improvement) improvement

^c^“Yes” is specified as report of any symptom improvement in the interview

^d^Degree is specified as report of no, mild, moderate or high improvement in the interview

**Table 3:** Bivariate correlations of symptom improvement variables in the **open-label** condition

Symptom improvement			IBS-SSS		Interview	
			Yes/No^a^	Degree^b^	Yes/No^c^	Degree^d^
IBS-SSS	Yes/No^a^	Spearman’s rho	1	.551	− .149	− .276
		*p* value		.079	.662	.411
	Degree^b^	Spearman’s rho	.551	1	.329	.161
		*p* value	.079		.324	.637
Interview	Yes/No^c^	Spearman’s rho	− .149	.329	1	.742
		*p* value	.662	.324		.009
	Degree^d^	Spearman’s rho	− .276	.161	.742	1
		*p* value	.411	.637	.009	

Sample size n = 11

IBS-SSS = irritable bowel severity scoring system

^a^ “Yes” is specified as at least 50 points of improvement on the IBS-SSS

^b^Degree is specified as no (< 50 points improvement on the IBS-SSS), mild (≥ 50 points improvement), moderate (≥ 100 points improvement) or high (≥ 150 points improvement) improvement

^c^ “Yes” is specified as report of any symptom improvement in the interview

^d^Degree is specified as report of no, mild, moderate or high improvement in the interview

#### Findings: attribution of improvement

While all participants in the double-blind condition who reported that their symptoms had improved during the trial thought that this improvement was probably or even certainly due to the treatment, there was much more variance in the open-label condition. Only 4 open-label participants thought that their improvement was probably or certainly due to the treatment, while 5 thought it was probably or certainly due to other reasons (Table 4). This difference between the two groups is statistically significant (*Χ*^2^[3] = 8.28; *p* = 0.041) and indicates that OLP participants were hesitant to attribute their improvement to such a paradoxical treatment.

**Table 4:** Attribution of symptom improvement in the two treatment conditions

	Certainly other reasons	Probably other reasons	Probably treatment	Certainly treatment
Open-label	1	4	1	3
Double-blind	0	0	4	7

The attribution categories were specified by quantitizing interview data. Only participants are included who reported any symptom improvement in the interview. The difference between the two groups is statistically significant (*Χ*^2^[3] = 8.28; *p* = .041)

## Discussion

The qualitative data of this study were assessed at the end of a six-week RCT investigating placebo effects in irritable bowel syndrome (IBS) that included both an open-label placebo (OLP) and a double-blind placebo (DBP) condition. While OLP participants were aware that they were receiving placebos, DBP participants did not know whether they received placebo or peppermint oil to treat their symptoms. To explore their experiences, we interviewed participants from both placebo treatment arms after they had finished the trial. In our interviews, the DBP group included participants of the peppermint arm as they were unaware of their group assignment and there was no different between placebo and peppermint. Two major themes of interest were identified in our qualitative analysis of the interviews: the participants’ feelings about their group allocation and their reflections about their treatment during the course of the study. In addition, we compared the participants’ narratives about their symptom improvement to their quantitative data on IBS severity that was assessed within the parent trial. To the best of our knowledge, this paper is the first to present comparative findings about participants’ experiences on OLP and DBP treatment and adopt both a qualitative and a mixed methods approach.

When comparing the two placebo groups qualitatively, we found interesting similarities within both major themes. The majority of the interviewed OLP and DBP participants were pleased with their treatment allocation and felt glad that they had not been randomized to the no-pill control group. Without being prompted, many of them told us they had been hopeful and curious regarding their treatment. The finding of hope as a major motivator to participate in a clinical trial—that includes the chance to receive placebo—is in line with our findings from a previous qualitative study of interviews with blind placebo participants [8]. However, our results demonstrate that the role of hope is not unique for participants in double-blind conditions but is mentioned in the same way by OLP participants, who know for certain that they will receive placebos. This may be related to the fact that our participants had previously visited many physicians and gastroenterologist without success. The subjunctive ‘what if it helps?’ stance thus seems to be central to both OLP and DBP participants. At the same time, curiosity toward the treatment has, to the best of our knowledge, not yet been reported to drive participants of placebo groups. In our study, curiosity seemed to be at least one reason for participants to join and finish the trial—no matter which condition they were allocated to. Arguably, the novelty of the idea that honest placebos could be a treatment in and of themselves triggered their inquisitiveness. Most interviewed participants were aware of the “power of placebo effects,” and participants of both treatment groups acknowledged that the placebo effect could have contributed to their symptom improvement. It is important to note, however, that positive treatment expectations were not mentioned explicitly by our interviewed participants—neither in relationship to their initial attitudes when entering the trial nor to their final interpretation of their experience.

We also found differences between the two placebo groups. These were especially evident in the language employed by OLP and DBP participants. While DBP participants tended to be enthusiastic about their treatment allocation and used predominantly strong and positive wordings to describe their feelings, OLP participants were more ambivalent. Their expression of neutral, inconsistent or even conflicting feelings toward their treatment reflects their cognitive dissonance and uncertainty on how to deal with the counter-intuitiveness of OLP.

The groups also differed in their thoughts and reflections about their treatment during the course of the study. Most DBP participants took a relatively passive stance towards the treatment, waiting for the effects of the pills and wondering whether they were on peppermint or placebo. Their attribution of symptom improvement was more straightforward—if they felt better they were sure to be on peppermint, and if they did not feel better, their guess was placebo. This was also supported by our quantitized data which demonstrated that all interviewed DBP participants who felt symptom improvement thought this was probably or even certainly due to the treatment. By contrast, we noticed that many OLP participants were more actively engaged with their treatment, consciously reflecting about its counterintuitive nature. Facing the paradoxical conundrum of being offered inert pills to treat their symptoms made them seriously reflect and contemplate not only about placebo effects but also about their own symptoms, their habits and their personal influence on how they feel. If their symptoms improved during the study, identifying the cause was not as simple as in DBP. Indeed, OLP participants offered a wide variety of possible explanations. Again, this was supported by our mixed data which showed that OLP participants had different ideas about the reason for their improvement. Only if participants did not feel any improvement, they were quick to attribute this to the inertness of their pills. This indicates that benefitting from OLP is often accompanied by flexibility in thinking and being involved in OLP may initiate flexible thinking processes. Participant 174 put it in a nutshell: *“Even if psychologically, I’m doing something to make myself feel better or I’m being proactive, that could have an impact.”* The conclusion of flexible thinking as a potential predictor or accompaniment of OLP effects aligns with recent theoretical work stressing the ability to shift cognitive sets as a therapeutic factor in itself [20].

Interestingly, our mixed data shows that only in DBP but not in OLP, the participants’ objective symptom improvement (as assessed during the quantitative parent trial) is in line with their narrative of the interviews. In other words, when asked whether they felt better and to what degree, the answers of DBP participants were highly correlated with their questionnaire data on IBS improvement—but the answers of OLP participants deviated substantially from their questionnaire scores. It appears that DBP participants were making specific efforts to monitor symptom changes out of curiosity regarding their treatment allocation whereas themes of symptom monitoring were not evident in the OLP interviews. This finding is in line with recent experimental evidence of OLP and DBP analgesia in healthy subjects [21]. In this experiment, OLP was as effective as DBP in reducing objectively measured pain tolerance, but this was not reflected by the participants’ subjective rating of pain intensity and unpleasantness, where only DBP participants indicated that their treatment had an analgesic effect. We hypothesize that attributing success to treatment without “active ingredients” may involve breaking with common sense and rational thinking, which is probably not easily embraced by research participants. Furthermore, patients may have resisted potential stigmatization and been unwilling to endorse that their IBS “was in their heads” and could be improved by a simple placebo [22]. This suggests that physicians who might adopt OLP in clinic need to better inform patients on the possibility of non-conscious embodied processes that could activate placebo effects [1, 23]. In this context, it may be helpful to explain to patients that improving from OLP is a sign of a flexible and healthy central nervous system, which can non-consciously adjust pain signals towards reduced symptoms and decrease neural excitability in therapeutic situations.

Our data suggest that patients treated by two different placebo models (DBP vs. OLP) share some similar experiences (hope, curiosity, awareness of placebo effects) but also have distinct experiences (enthusiasm vs. ambivalence, passive vs. active engagement). These qualitative findings support an emerging picture that, on multiple analytic levels, different placebo treatments utilize different mechanisms. For example, in our published parent study we found that higher baseline expectations predicted higher double-blind placebo responses [12], which is in line with the broad literature body of the last decades [24]. Dramatically, in that same study, we found the opposite in open-label placebo (higher baseline expectations predicted *lower* placebo effects). Genetic data also seem to suggest differences. Two recent studies of OLP genetics examined genetic variations in Catechol-O-Methyltransferase (COMT) polymorphisms (rs4680 and rs4818) and found that in OLP treatment rs4680 high-activity G-allele (G/G or G/A) or rs4818 C/G genotypes reported significant more improvement [25, 26]. In double-blind placebo RCTs, participants homozygous for the rs4680 A allele (A/A) were more likely to respond than G/G or G/A participants, the reverse of OLP [27]. These findings suggest that even the genetic association with treatment response may be different for OLP and DBP. Furthermore, emergent neuroimaging evidence in OLP suggests that “unlike other placebo effects, placebo analgesia without expectation [OLP] for pain relief may primarily engage lower order pain control mechanisms without prefrontal support” [28, 29].

### Limitations

Our findings provide a first impression of participants’ distinct experiences on OLP treatment as compared to DBP treatment. Due to the qualitative nature of this investigation, reliability and validity are limited, and subsequent quantitative studies are needed to further explore and eventually substantiate our findings. As the quantitized variables derived from our interview data may involve a risk of bias, future studies should include quantitative instruments to measure the participants’ personal evaluation of their symptom improvement. Also, our mixed methods analysis lacked a formal a priori power calculation. We took advantage of the availability of data to perform what should be considered an exploratory study, but the small sample size limits any conclusions drawn from the mixed methods analyses and must be considered when interpreting the findings. Furthermore, we decided a priori to interview only five treatment as usual (TAU) participants which gave us insufficient data for interpretation. Additional interviews with TAU participants (as well as potential no-treatment participants) would have been helpful to compare their experiences to the experiences of placebo participants. Moreover, the parent RCT involved two different placebos; it is possible that participants’ experiences would be different in RCTs and clinical practice with only an OLP option. Further qualitative studies with OLP could address this limitation. Even though the enteric coating of the peppermint pills is designed to eliminate smells, some of our participants in the double-blind condition might have detected a slight peppermint taste if they accidentally bit the capsules or experienced reflux after the intake. Although our team was not able to detect any odor of the capsules, we should have assessed blinding in our parent study on the assumption that any unblinding of the peppermint treatment might have increased positive responses to the active intervention. Nonetheless, we believe this possibility is unlikely, as data from our secondary paper comparing double-blind peppermint to double-blind placebo showed no difference in peppermint oil and placebo outcomes [17]. Finally, since we only interviewed our participants at the end of the RCT and not at the beginning, our conclusions about cognitive and emotional processes they underwent during participation are drawn retrospectively. This needs to be considered when interpreting our findings.

## Conclusion

The results of this qualitative mixed methods study suggest that hope and curiosity may be central for both DBP and OLP treatment. At the same time, OLP participants expressed more ambivalent feelings and were more actively involved with their treatment—in the sense of more substantial cognitive or emotional self-reflection—as compared to DBP participants. However, only in the DBP group, the participants’ retrospective report of their symptom improvement was correlated to their corresponding quantitative questionnaire data, while this was not the case for OLP. It seems that attributing benefits to placebo treatment is challenging for OLP participants, especially when they may also be thinking about the option of another active intervention (peppermint). Also, for many patients with functional diseases, responding to placebo may retain the old stigma of “it’s all in your head.” [22]. Our findings add to a recently emerging, but still incomplete picture of a variety of different mechanisms that may be involved in different placebo conditions and that may distinguish OLP and DBP effects.

## Data Availability

The datasets used and/or analyzed during the current study are available from the corresponding author on reasonable request.

## Abbreviations

OLP: Open-label placebo
DBP: Double-blind placebo
TAU: Treatment as usual
RCT: Randomized-controlled trial
IBS: Irritable bowel syndrome
IBS-SSS: IBS Severity Scoring System
COMT: Catechol-O-Methyltransferase
